# Difficulties in Defining Oligometastatic Prostate Cancer: Implications for Clinical Trial Accrual and Community Practice Adoption of Metastasis-Directed Therapy Approaches

**DOI:** 10.3390/jcm12052011

**Published:** 2023-03-03

**Authors:** Tanya Barauskas Dorff, Saro Kasparian, Natasha Garg, Sandy Liu, Sumanta Kumar Pal, Jeffrey Wong, Savita Dandapani

**Affiliations:** 1 Department of Medical Oncology and Therapeutics Research, City of Hope Comprehensive Cancer Center, 1500 E. Duarte Rd. Pavillion #2250, Duarte, CA 91010, USA; 2 Department of Medical Oncology and Therapeutics Research, City of Hope, Upland, CA 91786, USA; 3 Department of Medical Oncology and Therapeutics Research, City of Hope Lennar Cancer Center, Irvine, CA 92618, USA; 4 Department of Radiation Oncology, City of Hope Comprehensive Cancer Center, Duarte, CA 91010, USA

**Keywords:** oligometastatic, prostate cancer, metastasis-directed therapy, imaging, PSMA PET

## Abstract

Background: Metastasis-directed therapy is widely utilized for oligometastatic prostate cancer patients, but standard imaging does not always identify metastases definitively and, even with PSMA PET, there may be equivocal findings. Not all clinicians have access to detailed imaging review, particularly outside of academic cancer centers, and PET scan access is also limited. We sought to understand how imaging interpretation impacted recruitment to a clinical trial for oligometastatic prostate cancer. Methods: IRB approval was obtained to review medical records from all patients screened for the institutional IRB-approved clinical trial for men with oligometastatic prostate cancer involving androgen deprivation plus stereotactic radiation to all metastatic sites, as well as radium223 (NCT03361735). Clinical trial inclusion required at least one bone metastatic lesion and no more than five total sites of metastasis, including soft tissue sites. Tumor board discussion records were reviewed, along with results from additional radiology studies ordered or confirmatory biopsies performed. Clinical characteristics such as PSA level and Gleason score were studied for association with likelihood of oligometastatic disease confirmation. Results: At the time of data analysis, 18 subjects were deemed eligible and 20 were not eligible. The most common reasons for ineligibility were no confirmed bone metastasis in 16 patients (59%) and too many metastatic sites in 3 (11%). The median PSA of eligible subjects was 3.28 (range 0.4–45.5), whereas the median PSA of those found to be ineligible was 10.45 (range 3.7–26.3) when there were too many metastases identified, and 2.7 (range 0.2–34.5) when metastases were unconfirmed. PET imaging (PSMA or fluciclovine PET) increased the number of metastases, while MRI resulted in downstaging to non-metastatic disease. Conclusions: This research suggests that additional imaging (i.e., at least two independent imaging modalities of a possible metastatic lesion) or tumor board adjudication of imaging findings may be critical to correctly identify patients appropriate for enrollment in oligometastatic protocols. This should be considered as trials of metastasis-directed therapy for oligometastatic prostate cancer accrue and results are translated to broader oncology practice.

## 1. Introduction

Metastatic prostate cancer remains incurable despite recent improvements in outcomes with systemic therapy. Whereas previously any metastatic disease, even if only to pelvic lymph nodes, was felt to represent a disseminated disease state which should only be treated with systemic therapy, sophisticated analysis of metastases from an autopsy series identified that metastatic deposits can create additional metastases [1]. This raised the possibility that enhanced eradication of cancer at visible metastatic foci using focal radiation could prevent or reduce further cancer spread. Preliminary success with this approach has been observed. For instance, the ORIOLE trial [2] found improved progression-free survival when stereotactic ablative radiation (SABR) was used to treat oligometastatic sites (compared to observation). However, this study utilized PET scans to define oligometastases, and 19% of those treated with SABR had progression within 6 months, suggesting that additional occult metastatic sites existed.

Advances in imaging with prostate-cancer-specific PET tracers such as [^11^C]Choline, [^18^F]DCFPyL, and [^68^Ga]Ga-PSMA-11 have resulted in improved sensitivity and specificity for detecting metastatic foci [3,4,5]. However, these are not widely available, and the majority of prostate cancer patents enrolling on clinical trials continue to undergo conventional imaging with CT and technetium bone scans to define their eligibility.

Our institution is recruiting subjects to an IRB-approved investigator-initiated clinical trial (NCT03361735) designed to enroll men with oligometastatic, castration-sensitive prostate cancer. Participants receive 9 months of androgen deprivation therapy, stereotactic ablative radiation to all metastatic sites, and 6 doses of radium223 (55 kBq/kg intravenously, once every 4 weeks × 6 doses). The protocol requires at least one bone metastasis and no more than five sites of metastasis for inclusion. During screening for the clinical trial, it was noted that review of imaging by the tumor board, or additional imaging studies ordered to verify the oligometastatic status, often led to a determination that the patient was ineligible. Based on this observation, we obtained IRB approval to evaluate the subjects who did not successfully enroll, with the goal of evaluating which imaging modalities were most helpful in confirming or refuting an oligometastatic state and determining whether any clinical characteristics should raise questions about the certainty of imaging findings. This information was felt to have the potential to benefit future clinical trials for oligometastatic prostate cancer patients, and also help practicing oncologists note imaging pitfalls when recommending metastasis-directed therapy for patients presenting to them with what appears to be oligometastatic disease.

## 2. Methods

After IRB approval via an amendment to the main clinical trial protocol, a retrospective chart review was performed on patients who signed consent for the clinical trial protocol (NCT03361735) or were being considered for enrollment based on tumor board records.

Treatment on the trial included androgen deprivation therapy for 9 months, SBRT to all metastatic sites, and radium223 infusions (55 kBq/kg IV, once every 4 weeks for 6 doses). The primary endpoint of this trial was time to treatment failure. 

Baseline disease characteristics and the results of all imaging studies and biopsies that were performed as part of the eligibility determination were tabulated. For the purposes of this analysis, eligible men included those enrolled on the protocol, as well as those who were deemed eligible but declined participation, while ineligible men were those who were excluded from participation due to having more than the allowed number of metastases or a lack of confirmed metastatic disease after tumor board review or additional imaging or biopsy. Comparison between eligible and ineligible patient groups was performed using a *t*-test for continuous variables and chi-square for categorical variables.

## 3. Results

This study began in 2018. At the time of analysis, 18 subjects had been deemed eligible, while 20 others were deemed ineligible. Baseline characteristics are summarized in Table 1. The median PSA of eligible subjects was 3.28 (range 0.4–45.5), whereas the median PSA of those found to be ineligible was 10.45 (range 3.7–26.3) when there were too many metastases identified, and 2.7 (range 0.2–34.5) when no metastases could be confirmed. There was no difference in Gleason grade group between patients confirmed to be oligometastatic and those recategorized as either non-metastatic or having more than five metastatic sites.

MRI was performed in 19 of the 38 patients (50%) and PET scans were performed in 21 of 38 patients (55%). Biopsy of a bone lesion was performed in four cases (one each: femur, iliac, ischium, and rib), and three of these patients had PSA <1 at the time. In all four cases, biopsy was negative for malignancy, which was used to determine that the patient was ineligible. One of these patients later developed metastasis in a different bone but did not appear to develop metastasis in the original biopsied area. Three subjects with PSA >10 who were initially suspected of having metastatic disease were deemed non-metastatic after tumor board imaging review and/or MRI. Two subjects with PSA <5 were found to have too many metastases to qualify, in both cases based on fluciclovine PET imaging.

Skull (*n* = 2) and femur (*n* = 3) findings were most commonly recategorized as non-metastatic on further imaging or further review by the tumor board, while acetabular lesions were more commonly confirmed (*n* = 2). Spine and pelvic findings were evenly divided between confirmed and unconfirmed patients.

## 4. Discussion

Metastasis-directed therapy holds significant promise for oligometastatic prostate cancer, but clinical trials in this space have utilized different imaging modalities to define their oligometastatic populations (Table 2). In this experience, there was a high ineligibility rate during screening for a therapeutic clinical trial for patients with oligometastatic prostate cancer, with subjects having either too many metastases or a lack of confirmed metastases. This was the result of a high degree of scrutiny and utilization of additional imaging and/or biopsy in order to confirm eligibility. It raises questions about how community oncologists can best adopt metastasis-directed therapy for oligometastatic prostate cancer patients without access to the resources available at a tertiary academic center.

Variability in radiologic interpretation in cancer patients has been well documented, for instance when evaluating the RECIST response [8] and even when using conventional imaging, which most radiologists have the greatest amount of experience in interpreting. The lack of sensitivity and specificity of conventional imaging for identifying prostate cancer metastases has also been well documented [9]. For instance, in the POPSTAR trial, even with [^18^F]-NaF PET bone scans, there was considerable understaging as distant progression-free survival (PFS) was about 40% at 2 years compared to 89–100% continued remission at the sites of irradiation [6]. This indicates that smaller deposits of disease had not been visible when metastasis-directed therapy was administered.

PSMA PET tracers significantly improve sensitivity [3]. However, due to limited access and difficulty in interpretation, the research community has largely opted to continue basing eligibility and response assessment on conventional imaging. In the ORIOLE trial, for example, conventional imaging formed the basis for treatment. The protocol specified that [^18^F]DCFPyL-PET images were evaluated and compared to bone scans, but additional sites of suspected metastatic disease from the PET scan were not considered for treatment by SBRT nor required to undergo further evaluation [2]. This design resulted in the ability to analyze outcomes in patients whose PET-detected disease was fully treated (i.e., PET scan did not detect additional sites of disease beyond what was visible on conventional imaging) compared to those in whom some metastatic disease was left untreated, and it was noted that the former group had greater progression-free survival. Thus, future oligometastatic protocols are likely to rely on PSMA PET imaging. However, false positives will continue to be an important consideration since benign conditions such as Paget’s disease have been reported to result in false-positive PSMA radiotracer uptake [10], and interpretation can be challenging for this relatively newer imaging modality.

Access to PET scans for prostate cancer patients remains a major limitation in community practice and in academic centers. A recent publication found that in a tertiary medical center, there were disparities in PET scanning, with African American prostate cancer patients less likely to undergo PSMA PET scan compared to non-Hispanic white patients [11]. In the community oncology setting, differences in health insurance coverage and imaging facility capabilities may exacerbate the lack of equitable access to PET imaging. Community oncologists may also have decreased access to multidisciplinary care via participation in tumor boards. In one survey of community practices, 53.8% of physicians reported participating in tumor boards weekly, while 42% participated less than once per week, with less attendance from medical oncologists compared to radiation oncologists [12]. In our experience, tumor board review was key in gaining confidence for inclusion of patients and treatment of oligometastatic sites, even if PET imaging was not available, and lack of access to optimal imaging should not preclude patients from accessing the potential benefit of metastasis-directed therapy.

While a major focus has been placed on determining the number of metastatic lesions that define the oligometastatic disease state, it seems that controversy surrounds whether a conventional imaging modality can adequately establish a patient as having the specified number of metastases. Bone scanning has been suggested to yield inconclusive results in about 16% of cases [13]. MRI has often been used to enhance detection of osseous metastases or clarify inconclusive bone scan findings. However, in a study of findings from pelvic MRI performed in 3765 patients for evaluation of presumed localized prostate cancer, 74% of patients had bone abnormalities, which were only rarely confirmed to be metastases [14]. This calls into question the use of MRI to adjudicate findings from conventional imaging. Bone biopsies may be helpful in confirming a bone metastasis, but even in experienced centers with specific protocols designed to maximize the yield, the detection rates from bone biopsy performed to obtain cancer tissue have been reported to be less than 80% [15], and in most community centers without expertise the yield will be lower. Therefore, bone biopsy may not have a high enough sensitivity to be used to exclude the presence of metastatic cancer. Some clinical characteristics may be helpful in selecting metastases for greater yield, including the size of the lesion in the bone, presence of a soft tissue component, intensity of scintigraphic uptake, or a newly apparent area of disease involvement, but without communication between oncologists and radiologists, optimal target selection is less likely to occur.

Overall, our experience raises concern about a potential lack of uniformity in the population of patients who are subject to protocols for oligometastatic prostate cancer, and how the results can subsequently be translated into clinical practice. There is no defined algorithm for how to confirm oligometastases identified in a prostate cancer patient using conventional imaging. We found that simple clinical factors may help guide clinicians as to when additional scrutiny is warranted. In this research, subjects with PSA over 10 were less likely to be deemed oligometastatic after further imaging or imaging review, although there were four subjects who were found eligible with PSA ranging from 10 to 45. Similarly, subjects were less likely to have metastases at all when PSA was less than 1, though four subjects were deemed to have oligometastatic disease at this PSA level, only one of whose disease was detected on a PET scan. While clearly not enough to define an oligometastatic state, higher or lower PSA should at least raise clinical suspicion and trigger additional imaging, consultation with radiology, or potentially a biopsy to better clarify the extent of the disease. Where PSMA PET scans are not available, any indeterminate bone findings, or a discordance between bone scanning and CT, or between the volume of disease and PSA, may warrant additional imaging and/or biopsy before metastasis-directed therapy is undertaken.

## 5. Conclusions

Defining the oligometastatic state depends on accurate interpretation of imaging. In our experience, prostate cancer patients initially thought to be oligometastatic were frequently reclassified when additional imaging was ordered to clarify indeterminate findings. Physicians should be encouraged to thoroughly review imaging, utilizing tumor boards or additional imaging modalities when appropriate, prior to applying metastasis-directed strategies for their patients who appear to have oligometastatic prostate cancer.

## Figures and Tables

**Table 1:** Characteristics of eligible and ineligible patients being evaluated for enrollment into the oligometastatic protocol.

	Eligible (*n* = 19)	Ineligible—Too Many Metastases (*n* = 6)	Ineligible—Not Metastatic (*n* = 13)	*p* Value ^
Median PSA * (range)	3.28 (0.4–45.5)	10.45 (3.7–26.3)	2.7 (0.2–34.5)	Many *p* = 0.057 Few *p* = 1.0
Primary untreated	18.1 (9.1–45.5)	8.9 (4.6–13.1)	13.7 (4.8–34.5)	
Primary treated	1.7 (0.4–27.5)	12 (3.7–26.3)	1.174 (0.2–2.3)	
Gleason grade group N (%)				
1	3 (16%)	0	1 (8%)	*p* = 0.17
2–3	8 (42%)	4 (67%)	4 (31%)	*p* = 0.37
4–5	8 (42%)	2 (33%)	8 (61%)	*p* = 0.14
Imaging modalities				
MRI	10	1	7	
PET (fluciclovine)	7	4	5	
PET (PSMA)	3	0	1	

* PSA at the time of eligibility assessment for enrollment on the clinical trial. ^ *p* values were calculated comparing eligible patients to those with too many (“Many”) metastases and eligible patients to those with too few (“Few”) metastases using Fisher’s exact test for continuous variables and the chi-square test for the Gleason grade group comparing too many versus too few.

**Table 2:** Imaging used to qualify patients for enrollment in select published oligometastatic prostate cancer clinical trials.

Study	# Metastases	Other Restrictions	Imaging Used to Define # of Metastases
POP-STAR [6]	1–3	Bone or LN only	[^18^F]-NaF PET/CT
ORIOLE [2]	1–3	Asymptomatic, arose in the prior 6 months, ≤5 cm in long axis or ≤250 cm^2^	Conventional imaging
STOMP [7]	1–3	Extracranial, negative MRI or biopsy of prostate bed even if choline PET negative in prostate bed	[^11^C]Choline PET

# signifies the number of metastases.

## Data Availability

Data sharing not applicable. This study is still accruing patients. Data sharing is not applicable to this article since full data from the clinical trial will be shared upon completion of the study.
